# Preparation and Application of Hydrophobic Film-Coated Recycled Low-Iron Crushed Glass via SiO_2_–Mixed TiO_2_ Through Sol–Gel for Efficient Removal of Water Contaminants Photocatalytically

**DOI:** 10.3390/toxics14040304

**Published:** 2026-03-31

**Authors:** Jiaxin Liu, Saeed Rad, Junfeng Dai, Cheng Chang, Chongmin Liu

**Affiliations:** 1 School of Environmental Science and Engineering, Guilin University of Technology, Guilin 541004, China; 2 Guangxi Key Laboratory of Environmental Pollution Control Theory and Technology, Guilin University of Technology, Guilin 541006, China

**Keywords:** nano TiO_2_, SiO_2_ mixture, sol–gel, hydrophobic coating

## Abstract

Traditional nano-titanium dioxide films have strong photocatalytic performance; however, their hydrophilic surfaces make it easier for pollutants or by-products resulting from the reaction processes to deposit on the membrane surface and occupy their active sites, which reduces the coating degradation efficiency and shortens their service life. In the current study, nano-TiO_2_ was mixed with SiO_2_ for hydrophobic film coating by the sol–gel method. The surface morphology of the membrane was observed by scanning electron microscopy (SEM), the composition of the coating was analyzed by X-ray diffraction (XRD), and its stable hydrophobicity was verified by contact angle testing (θ_w_ = 117°). The specific surface area Brunauer–Emmett–Teller (BET) revealed between 0.0561 (for 3 layers) and 0.0868 m^2^/g after 9 layers of coating. Through establishing a simplified photocatalytic reactor under UV, the new coating’s abilities in the degradation of methylene blue, its anti-fouling, and durability were examined. Results revealed that when the common TiO_2_ films were combined with hydrophobic films, nearly 100% of methylene blue was degraded, and the degradation capacity remained stable after three rounds of tests. Moreover, it was observed that only a small amount of methylene blue adhered to the new film surface comparatively. Outcomes confirmed that the SiO_2_-mixed TiO_2_ thin films exhibited enhanced hydrophobicity. When integrated with ordinary TiO_2_ coatings, the composite structure demonstrated superior photocatalytic efficiency and stability in the degradation of aqueous pollutants compared to pure TiO_2_ coatings.

## 1. Introduction

Water quality is of vital importance and plays a key role in maintaining biological, social, and economic systems. It can be said that the quality of water determines the quality of life. Over the past few decades, along with the rapid development of human civilization, the causes of water pollution have become increasingly diverse as society progresses. Serious water pollution due to various human activities such as industrial waste discharge, mining activities, the use of pesticides and fertilizers, energy consumption, and radioactive waste discharge has led researchers to constantly explore more efficient, lower-cost, and more environmentally friendly water treatment methods [1,2]. As a result, a wide variety of treatment methods have emerged in an endless stream. Traditionally, several physical, chemical, and biological technologies, such as flotation [3], precipitation [4], oxidation [5], solvent extraction [5], evaporation, carbon adsorption [6], ion exchange [5], membrane filtration [7], electrochemical methods, biodegradation [8], etc., have been reported. Some of these methods have made significant progress in removing harmful compounds from water. However, recent advancements, such as ultrasonic oxidation, membrane technology, and the application of cations in nanomaterials, especially photocatalytic degradation [9] and advanced oxidation processes (AOPs) [10,11], have attracted the attention of researchers. Among the AOPs, nano-titanium dioxide is a material with great potential in this field. It has been commercialized in a variety of industries such as construction [12], medical [13,14], food antibacterial [15], cosmetics [16], and photoelectron-chemistry [17], just to mention a few. Nano-TiO_2_ has shown outstanding abilities in photocatalytic water treatment due to its remarkable catalytic performance, stability, high photocatalytic efficiency, low toxicity, and ability to degrade various pollutants [18,19].

The photocatalytic degradation principle of water pollutants is primarily based on the energy band structure of semiconductor materials. When nano-titanium dioxide is irradiated with a light having an energy greater than its band gap (3.2 e.V), excited electrons jump from the valence band to the conduction band, generating photogenerated electron–hole pairs. These pairs have strong oxidation–redox potential and react with water and oxygen adsorbed on the titanium dioxide surface to form hydroxyl radicals (·OH) and superoxide radicals (O_2_^−^), respectively [19,20]. The resulting radicals are highly reactive and can degrade organic or inorganic pollutants in water [21]. Numerous studies have shown that nano-TiO_2_ has photocatalytic properties under ultraviolet light (especially its anatase phase, since brookite and rutile phases are far less active photocatalytically) that can reduce bacteria [22], organic compounds [23,24], heavy metal ions [21,25], and dyes [26,27], etc. However, the fact is that during the photocatalytic reaction, fouling results in a reduction in the photocatalytic degradation efficiency, a phenomenon in which pollutants, intermediates, or by-products of the reaction process will be deposited on the surface of the nano-titanium dioxide, occupying the active sites and causing the reaction rate to decrease. This is a critical point that cannot be ignored in using nano-titanium dioxide to degrade pollutants in scale-up reactors.

Conventional nano-titanium dioxide films are mostly hydrophilic, which makes the coating vulnerable to absorbing pollutants in water or by-products in the reaction process, to deposit on the film surface. As a result, the fouling significantly increases the maintenance cost of the film or will lead to recoating. Therefore, hydrophobic nano-titanium dioxide colloids can address this issue. In recent years, the development of hydrophobic self-cleaning surfaces with photocatalytic properties has attracted considerable attention. For instance, Ju Ha Lee et al. [28] mixed hydrophobic PDMS (polydimethylsiloxane, hydroxy-terminated)-coated silica nanoparticles with photocatalytically active N-doped TiO_2_ and distributed them on a glass substrate. They obtained a film with both hydrophobicity and photocatalytic activity through this method, and confirmed its ability to degrade pollutants and the persistence of photocatalytic activity in wastewater treatment due to hydrophobicity. Jie Liu et al. [29] controlled the thickness of the polymer layer between 0.6 and 5.5 nm by changing the molecular weight of PDMS used to modify the surface of TiO_2_-covered glass, thereby obtaining a film with both photocatalytic activity and hydrophobicity. Xu et al. [30] combined TiO_2_, polyvinylidene fluoride (PVDF), and PDMS and sprayed them on a glass substrate to form a superhydrophobic film. Their results show that the water droplets’ contact angle on the film slightly decreases when exposed to free air. Although the modification controls the surface wettability, it was concluded that this method can affect the photocatalytic performance of the surface. Ali Ansari et al. [31] used electrostatic spraying to prepare a hydrophobic photocatalytic composite coating based on nano-TiO_2_ and fluorinated ethylene propylene. This coating had a remarkable wear resistance, and they found that the higher the weight percentage of TiO_2_, the lower the static contact angle, which can be assigned to the hydrophilic properties of TiO_2_ nanoparticles. The above studies used various surface modifications to make the titanium dioxide films hydrophobic without markedly losing their photocatalytic activity; however, the photocatalytic performances are often affected.

In this study, SiO_2_-mixed TiO_2_ hydrophobic sol was prepared using PDMS (polydimethylsiloxane, hydroxy-terminated), TEOS (tetraethyl orthosilicate), and TTIP (titanium(IV) isopropoxide), and the obtained gel was coated on recycled low-iron crushed glass. This work aims to enhance the hydrophobicity and microporosity features of the conventional TiO_2_ coatings used for photocatalytic applications via a simple sol–gel method cost-effectively. PDMS is incorporated to enhance the hydrophobicity of the composite film. Concurrently, the SiO_2_ is mixed to facilitate the formation of a TiO_2_–SiO_2_ hybrid film characterized by a substantial number of microporous structures, which endows the material with a high surface area [32]. In order to obtain a coating with both hydrophobicity and photocatalytic activity, we first coated the support surface with ordinary nano-TiO_2_ gel several times and then with a hydrophobic gel as the last layer. The films were characterized using scanning electron microscopy (SEM), X-ray diffraction (XRD), BET, and contact angle measurements. In addition, comparative experiments using hydrophobic and conventional TiO_2_ films were conducted in a simplified water treatment reactor. Dye degradation experiments were performed to evaluate the photocatalytic performance and sustainability of the coating by monitoring the removal rate of dye pollutants over time.

## 2. Materials and Methods

### 2.1. Materials

The titanium(IV) isopropoxide (TTIP) (99.9%), diethanolamine (DEA) (99%), tetraethyl orthosilicate (TEOS) (99.99%), and polydimethylsiloxane (PDMS) (hydroxy-terminated) (65 cP) used in this study were all analytical grade, and provided by Shanghai Aladdin Biochemical Technology Co., Ltd., Shanghai, China while nitric acid (65%) and ethanol (99.9%) were mutually from Xilong Chemical Co., Ltd., Shantou, China.

### 2.2. Preparation of the Hydrophobic Coating

#### 2.2.1. Nano–SiO_2_TiO_2_ Gel Preparation via the Sol–Gel Method

The coating preparation in this study can be divided into two steps. The first step is to obtain the gels (ordinary TiO_2_ gel, and the hydrophobic nano-SiO_2_–TiO_2_ gel) through the sol–gel method, and the second is to coat the films on the support surface, which was carried out through the dip coating method.

In order to prepare about 780 mL of ordinary TiO_2_ gel, the following steps were taken in this study. First, 600 mL of anhydrous ethanol was prepared in a 1000 mL beaker on a magnetic stirrer at a rotating speed of 500 rpm. Subsequently, 60 mL of Ti precursor isopropyl titanate (TTIP) solution and 40 mL of stabilizer diethanolamine (DEA) were added in turn. The stirring process lasted for 1 h to ensure that the TTIP was completely dissolved in ethanol. Using a pear-shaped separation funnel with a capacity of 1000 mL, 80 mL of diluted hydrochloric acid (HNO_3_) with a pH of 1.1 was dripped into the solution, at a dripping speed of about 15 s/drop. After that, the mixture was continuously stirred for 48 h, and the solution transformed into a translucent sol. The beaker was then sealed with plastic wrap and left undisturbed in the laboratory for 96 h. Finally, the aging process transformed the sol into a gel, which was ready to be applied for coating.

**Figure 1:**
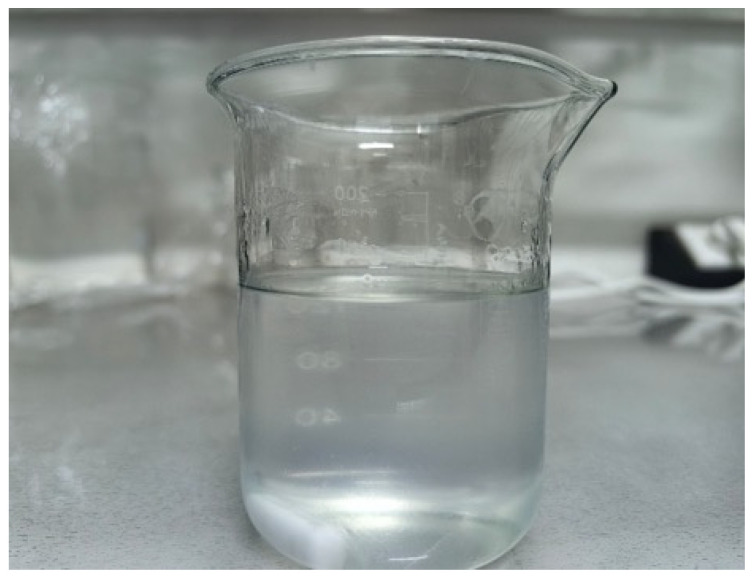
The clear viscos SiO_2_–TiO_2_ gel.

To obtain the SiO_2_–TiO_2_ sol, first, 20 mL of tetraethyl orthosilicate (TEOS) was added to a 100-mL beaker containing 45 mL of ethanol as the solvent in a dehumidifying environment (with the air conditioner at approximately 22 degrees Celsius). The mixture was then pre-hydrolyzed for 150 min under magnetic stirring at 500 RPM. Later, 3 mL polydimethylsiloxane (hydroxy-terminated) was added to the solution dropwise while stirring. After 15 min, 1.7 mL DEA and 2.5 mL TTIP were sequentially added. Following an hour of stirring, 1 mL of nitric acid was slowly dropped into the solution over a period of 40 min. The resulting solution was stirred at 500 RPM for about 20 h. Finally, it underwent an aging process for 20 h at room temperature, then the obtained translucent SiO_2_–TiO_2_ sol was converted into a clear viscous gel. The resulting gel is shown in Figure 1.

#### 2.2.2. Selection of Support

Nano-TiO_2_ powder is usually used in water treatments in either suspended or fixed forms. However, the suspended form may cause secondary pollution. In contrast, fixing TiO_2_ as a thin film on the surface of various carriers has become a more promising water treatment method. Different materials, such as glass [33,34,35,36,37], metal plates like stainless steel [38,39], tiles [27], and even some substrates with poor thermal stability, such as polymers [40], have been used as carriers for coating films applied in photocatalytic degradation and pollutant removal.

This study used low-iron crushed glass as the supporting material. The reasons for choosing low-iron crushed glass as the supporting material were its scalability, transparency, durability, heat resistance, UV resistance, and cost-effectiveness. In addition, the small size of the crushed glass and its relatively large surface area also impart significant advantages to the photocatalytic degradation of water pollutants.

#### 2.2.3. Film Coating Process

Before the coating process, cleaning the support surface is indispensable. For this purpose, first, the recycled low-iron crushed glass pieces (with an average diameter of 3–6 mm) were rinsed with ultrapure water for 10 min to remove any remaining glass powder, dust, or impurities from the production process. Then, they were transferred into a 1 L beaker and soaked in an alcohol solution, prepared by mixing ethanol and water in a ratio of 1:3 for 24 h. Next, the support materials were placed in an ultrasonic bath (F-050 SD, 15 L/300 W, Fuyang Technology Co., Ltd., Shenzhen, China) running at a frequency of 40 kHz for 15 min for ultrasonic treatment. After sonication, the supporting material was transferred to an oven and dried for 4 h at 105 °C. Finally, the crushed glass pieces were placed in a clean tray and sprayed with acetone (99.5%, Xilong Chemical Co.) to thoroughly clean and dry the support.

After the completion of these steps, 300 mL of gel was poured into a 500 mL beaker, and the crushed glass was submerged in the beaker until the scale line reached 350 mL. To ensure that every angle of the crushed glass was entirely covered with the gel, they were gently stirred in the beaker with a glass stirring rod for 5 min. Then, the mixture of gel and glass was filtered using a 150 mm-diameter filter funnel with a 1 mm mesh size and left undisturbed for 30 min to allow for complete drainage of the gel. Later, the coated glasses were transferred into a hydrophobic Teflon plate and dried in an oven at 105 °C for 4 h. Supports were coated with nine layers of gel as the optimum number of layers based on our previous research work [41]. Finally, the dried supports went through an annealing process in a muffle (OLABO SX2-4-10 G, OLABO Instrument Co., Ltd., Jinan, China) at 550 degrees Celsius for 2 h for crystallization and maximum anatase phase transformation. Since the hydrophobic SiO_2_–mixed TiO_2_ gel loses its hydrophobicity during the annealing process at high temperatures (PDMS, which plays an important role in the film hydrophobicity, will begin to vaporize and decompose above 277.5 °C [42]), the samples coated with the hydrophobic colloid did not undergo the annealing process, while the samples coated with ordinary gel did after every three layers of coating (a total of three annealing processes). For the 9-layer ordinary gels, lastly, a layer of hydrophobic gel was coated to achieve hydrophobic surface modification.

For the subsequent experiments on methylene blue removal, besides the uncoated support as a control, another three different types of coated support were prepared, transferred to the reactors, and labeled as:

1. Normal crushed glass (support) with no coating, labeled as NG;
2. Support coated with nine layers of ordinary TiO_2_ gel, labeled as 9LOG;
3. Support coated with nine layers of hydrophobic SiO_2_-mixed TiO_2_ gel, labeled as 9LHG;
4. Support that was first coated with nine layers of ordinary TiO_2_ gel and then with 1 layer of hydrophobic SiO_2_–mixed TiO_2_ gel, labeled as 9O1HG.

### 2.3. Photocatalysis Evaluation of the New Coating

In order to compare the photocatalytic degradation and fouling prevention capabilities of ordinary versus the hydrophobic titanium dioxide film, we conducted a dye degradation experiment based on the following process.

#### 2.3.1. Simplified Photocatalytic Reactor for Dye Degradation Experiments

**Figure 2:**
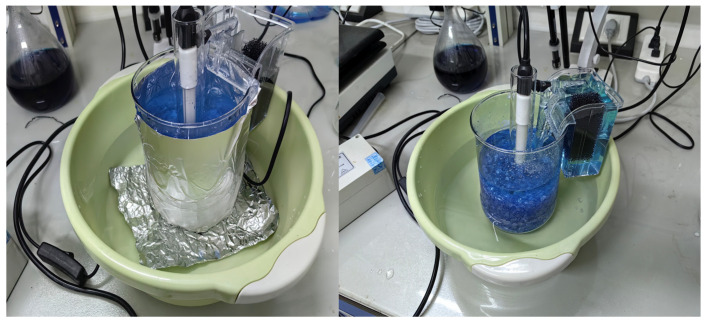
The simplified photocatalytic reactor designed for this study.

To make the photocatalytic reactor in this experiment, 1000 mL beakers were used to study the degradation efficiency of methylene blue. For this purpose, first, a 20 cm long quartz glass tube with a 4 cm inner diameter was placed in the center of the beaker, where the UV lamp (15 cm, 45 W, 365 nm) was inserted. This transparent tube (85% transparency to UVA) prevents the UV lamp from being damaged by the circulating polluted water. The crushed glass coated with different numbers of coated layers of TiO_2_ and SiO_2_–mixed TiO_2_ gel was transferred into the beaker until it surrounded and covered the UV lamp. Each reactor was filled with glass fragments up to the 600 mL mark, and then the pollutant solution was poured into the reactor until it reached the 1000 mL mark. Afterward, a small aquarium-type water pump with a plastic hose was hung on the edge of the beaker, while the other end of the hose could reach the bottom of the beaker. This pump was to ensure that the water in the reactor could circulate and maximize the possibility of pollutant and catalyst contact. An air pump was employed to continuously introduce oxygen to generate more active free radicals and enhance the photocatalytic efficiency. The beakers were covered with aluminum foil to reflect back the UV beam lights into the reactor, provide dark conditions for the control tests as well as for the safety of the examiners. The entire system was placed in a pan with cold water as a cooling bath to balance the heat generated from the UV. The explained reactor is presented in Figure 2.

#### 2.3.2. Selection and Preparation of Target Pollutants

Methylene blue, with the chemical formula of C16H18N3ClS, is a phenothiazine salt in the form of dark green bronze luster crystal or powder, soluble in water and ethanol. It undergoes a certain degree of self-degradation under ultraviolet light, and the methylene blue solution is easily adsorbed on the surface of various catalysts [43,44,45]. Methylene blue has the most extensive and rich data in the research on the photocatalytic degradation of dyes. It has a high degradation efficiency, a significant color fading effect, and is convenient for photometric monitoring. Moreover, the absorption degree of methylene blue on the catalyst surface can highlight the importance of the coating’s hydrophobicity for titanium dioxide films; therefore, this study used methylene blue as the target pollutant for degradation measurements. For comparison and control in our study, the degradation degree of this component under UV was measured by setting a control group under ultraviolet light in the absence of a catalyst.

When working with methylene blue, the concentration setting of the dye pollutant is an important parameter. If the pollutant concentration is too low, it will be impossible to distinguish the photocatalytic ability between the control group and the experimental group. Additionally, if the pollutant concentration is set too high, the solution color will be dark, which blocks the ultraviolet light from penetrating, resulting in a significant decrease in the photocatalytic reaction rate. After a series of initial tests, the concentration of the methylene blue solution was optimized at 20 mg/L, in which the photocatalytic results for the control and the experimental groups could be distinguished without a significant decrease in the photocatalytic reaction rate.

To prepare the polluted samples, methylene blue powder with a purity of 98% (Shanghai Macklin Biochemical Technology Co., Ltd., Shanghai, China) was used. A total of 0.1 g of methylene blue powder was dissolved in pure water and transferred to a 1000 mL volumetric flask to prepare a mother liquor of 100 mg/L. Then, 200 mL of the mother liquor was transferred into a 1000 mL volumetric flask to prepare 20 mg/L of contaminants.

To draw a standard curve, 5, 10, 15, 20, and 25 mL of 100 mg/L methylene blue were transferred to five 100 mL volumetric flasks with pipettes (from the mother liquor) to prepare solutions of 5, 10, 15, 20, and 25 mg/L, respectively. The absorbance of the contaminant was measured using a UV spectrophotometer(UNICO UV-2355, UNICO Instrument Co., Ltd., Shanghai, China). Finally, a standard curve between concentration and absorbance was established based on the measured data.

#### 2.3.3. The Experimental Process for the Removal of Dye Contaminants

Including UV as the second variable, we conducted five sets of treatments using the above-mentioned four types of glass fragments, such as:

1. Using uncoated normal crushed glass with no ultraviolet light exposure;
2. Using uncoated normal crushed glass under ultraviolet light exposure;
3. Using 9LOG under ultraviolet light exposure;
4. Using 9LHG under ultraviolet light exposure;
5. Using 9O1HG under ultraviolet light exposure.

For each test round, 20 mL of the solution sample was collected every two hours. During a total 16-h period, eight water samples were collected. Since the glass fragments (support) can absorb the dye on their surface to some extent, we conducted three rounds of experiments, adding a newly prepared 20 mg/L methylene blue solution in each round without changing the support. This technique was carried out to reach the dye absorption limit on the support and eliminate the influence of such phenomena as a variable. The concentration of dye pollutants before and after the test was measured via an ultraviolet–visible spectrophotometer (UNICO UV-2355, UNICO Instrument Co., Ltd., Shanghai, China). The wavelength was set at 665 nm to determine the absorbance of the sample. The absorbance peak of methylene blue in the ultraviolet–visible spectrum can be used to determine the concentration using Beer–Lambert’s law, as shown in Equation (1) [46]. The removal amount of methylene blue can be expressed by the removal rate and calculated using Equation (2) [47].

(1) A=εbC 

(2) %Removal=(Ci−Ct) /Ci×100

where *A* represents the absorbance, *ε* is the proportionality constant, *b* is the path length of the beam through the sample compartment, *C* indicates the concentration of methylene blue in the solution, while *C_i_* and *C_t_* show the initial concentration and concentration at time *t*, respectively.

This study also employed the removal rate research method, in which a linearized pseudo-first-order (PFO) kinetic model was used to determine the removal rate of MB. Through calculations based on Equation (3), the rate constant and half-life could be obtained using the PFO model.

(3) $$ln\left(qₑ-qₜ\right)=ln\left(qₑ\right)-k^{1}\times t$$

where *k* denotes the removal rate constant (/h), and *q*ₜ and *q*ₑ represent the removal capacity at time t and equilibrium, respectively. The value of *k* is proportional to the MB removal rate.

### 2.4. Coating Characterization

The conventional titanium dioxide films and the hydrophobic titanium dioxide films were characterized to determine their structures, morphologies, particle sizes, and surface properties. The structures of both types of films were analyzed by X-ray diffraction (X’Pert3 Powder-DY5103, PANalytical Co., Almelo, The Netherlands), and the surface morphologies of the three films were observed by field emission scanning electron microscopy (Gemini SEM 300, Carl Zeiss Co., Oberkochen, Germany) with an energy-dispersive X-ray (EDX) detector. The static contact angles of the two films before and after the photocatalytic tests were measured using a contact angle measuring instrument (DSA100, KRÜSS GmbH Co., Hamburg, Germany) to evaluate the hydrophobicity of the films.

## 3. Results and Discussion

### 3.1. Gel Characteristics

The TiO_2_ gels obtained in the laboratory were basically semi-transparent with a hint of white. The degree of whiteness and semi-transparency, as well as the viscosity of the same gel, are all usually related to the amount of solvent and the duration of aging. The more solvent used, the shorter the aging time, the more transparency, and the lower the viscosity, and vice versa.

**Figure 3:**
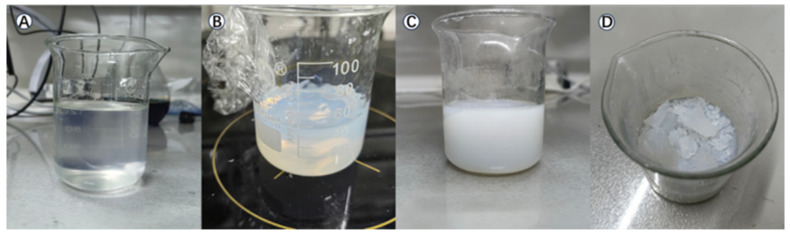
The gel and xerogel obtained: (**A**) low-viscosity hydrophobic gel, (**B**) high-viscosity hydrophobic gel, and (**C**,**D**) xerogel.

The attained gel must swiftly be used, since it cannot last long in its jelly form at room temperature, and will turn into xerogel after about 36 h. The main factors influencing the duration for which the gel stays in such form are temperature and air humidity. If it is stored in a low-temperature and dry environment (refrigerator), the gel state can be extended by approximately 3 to 4 days. However, if it is stored in a humid or warm environment, it will quickly change to the xerogel state. The gel and xerogel we obtained are shown in Figure 3.

### 3.2. Coating Characteristics

#### 3.2.1. The Surface Morphology of Coatings via SEM

**Figure 4:**
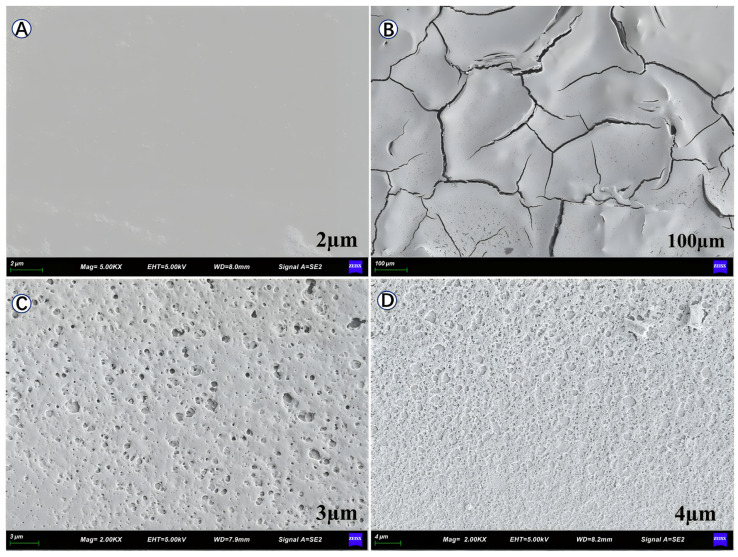
SEM images of the nano-TiO_2_ film: (**A**) the surface of a film prepared by coating a layer of low-viscosity hydrophobic gel, (**B**) the film prepared by coating a layer of high-viscosity hydrophobic gel, (**C**) the surface of the film prepared by coating nine layers of hydrophobic gel, and (**D**) the surface of the film prepared by coating nine layers of ordinary gel and then one layer of hydrophobic gel.

The coatings’ photocatalytic performance is closely related to the surface morphology and characteristics of the film; therefore, SEM tests were conducted on the titanium dioxide films to observe their surface morphology. For this test, four types of film samples were prepared; the first two were made using a layer of hydrophobic film prepared from the SiO_2_-mixed TiO_2_ gel on two flat glass substrates: one with a colloid of lower viscosity and another of higher viscosity. The third one was nine layers of film prepared using hydrophobic SiO_2_–mixed TiO_2_ gel on a flat glass substrate. The last sample was a layer of hydrophobic (SiO_2_–TiO_2_) film coated after nine layers of ordinary TiO_2_ film on a glass substrate, which was basically the same as the one used in the subsequent photocatalytic degradation experiment of methylene blue. The SEM images of the four samples are shown in Figure 4.

According to the SEM images, films coated with SiO_2_–mixed TiO_2_ gel of higher viscosity (Figure 4B) exhibited uneven flatness, while those coated with a gel of lower viscosity (Figure 4A) showed a smoother surface. During the drying process, the thicker parts of the uneven gel coating showed stress-caused changes because the top layer dried faster than the bottom layer. This drying time difference will result in cracks and the formation of peeling, which leads to the incompleteness and low quality of the final film produced, which will ultimately affect the photocatalytic ability and durability of the final product. This observation shows that in this process, using a gel with a high viscosity for film coating is not a recommended option.

In comparison, the glass substrate coated with nine layers of a hydrophobic film (Figure 4C as 9LHG) using the SiO_2_–mixed TiO_2_ gel displayed no significant cracking but instead possessed a uniformly distributed and finely porous architecture. Although this porous network may be mechanically less robust than a fully dense film, it is structurally superior to the cracked coatings. Moreover, the porous morphology offers a distinctive advantage over non-porous films as it significantly increases the effective contact area with aqueous solutions, thereby supplying more active sites for the photocatalytic degradation of water-borne pollutants and enhancing the overall degradation efficiency.

The surface morphology of the coating observed by SEM also provides an explanation for the different degrees of staining phenomenon observed among the four glass substrates after three methylene blue light catalytic degradation tests. The NG and 9LOG exhibited inherently hydrophilic surfaces. This characteristic promotes the spreading and adsorption of the aqueous methylene blue solution, leading to more pronounced staining.

In contrast, both 9LHG and 9O1HG demonstrated hydrophobic properties. However, a significant disparity in their residual staining levels is evident. Figure 4C,D reveals that this difference originates from their distinct surface morphologies. The 9LHG surface features a highly three-dimensional porous architecture as SiO_2_–mixed TiO_2_ gel. While this configuration increases the specific surface area, the deep, intricate pores can physically trap methylene blue molecules, facilitating dye retention and leading to comparatively heavier staining. Conversely, 9O1HG, benefiting from its composite layered design, presented a surface with a relatively flat topography. This morphology not only provides ample contact area for the photocatalytic reaction, but it also minimizes the deposition sites of methylene blue to the greatest extent possible.

**Figure 5:**
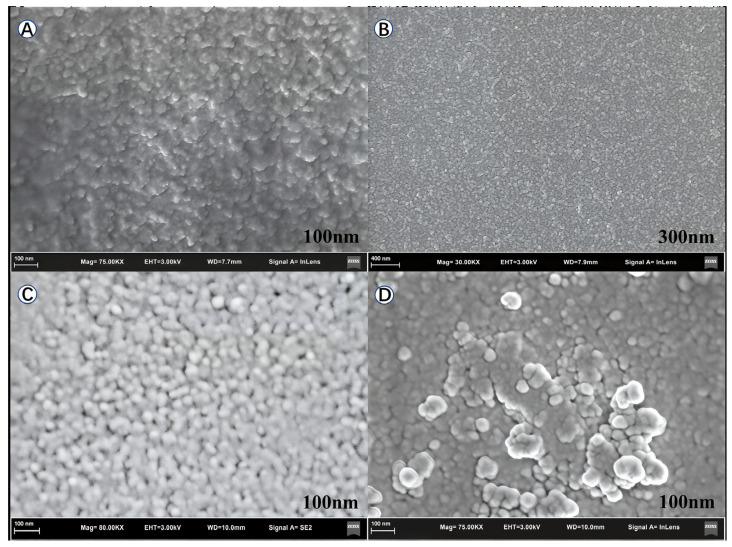
Nanoscale SEM images of nano-TiO_2_ film: (**A**) the surface of a film prepared by coating nine layers with hydrophobic SiO_2_–mixed TiO_2_ gel, (**B**) the surface of a film prepared by first coating nine layers of ordinary gel and then one layer of hydrophobic SiO_2_–mixed TiO_2_ gel, (**C**) the surface of the nine-layer film coated with low-viscosity ordinary gel, (**D**) the surface of the nine-layer film coated with high-viscosity ordinary gel.

The morphological characteristics and the nanoscale particle size of the TiO_2_ coating were analyzed more precisely using SEM images. More detailed SEM images of the four samples are shown in Figure 5.

The surface morphology of a film coated solely with nine layers of hydrophobic SiO_2_–mixed TiO_2_ gel is presented in Figure 5A. It features a non-uniform particle size distribution and considerable surface roughness, characteristics that are typically associated with hydrophobic surfaces. However, this elevated surface roughness can facilitate the accumulation of pollutant deposits, which in turn diminishes the coating’s durability and compromises its photocatalytic performance over multiple reaction cycles.

The surface morphology of the film, which was first coated with nine layers of ordinary gel and then with one layer of hydrophobic SiO_2_–mixed TiO_2_ gel, is shown in Figure 5B. As shown in this figure, on its surface, the nanoparticles were uniformly distributed, smooth, and flat, resembling the structure of standard high-quality films. When combined with subsequent hydrophobicity assessments, this uniform and compact structure suggests that the film successfully integrates durability, hydrophobicity, and sustained high photocatalytic efficiency across multiple operational cycles. This inference was subsequently corroborated by multi-cycle methylene blue degradation experiments.

Figure 5C demonstrates that film deposition using a low-viscosity gel yields a uniform and well-ordered nanoparticle arrangement on the surface. This homogeneous microstructure is indicative of a superior-quality coating, which corresponds to enhanced durability and higher photocatalytic performance. Figure 5D, in contrast, reveals that the film derived from a high-viscosity gel exhibits significant nanoparticle aggregation. This closely packed morphology reduces the effective surface area available for interaction with pollutant molecules and compounds, thereby compromising the film’s photocatalytic efficiency and long-term durability.

#### 3.2.2. XRD and BET Assessment of the Coating

**Figure 6:**
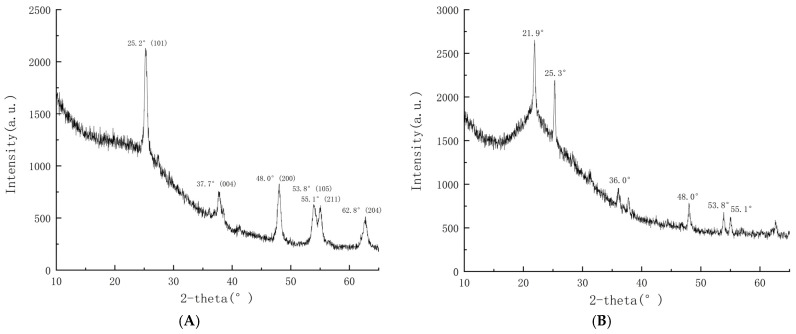

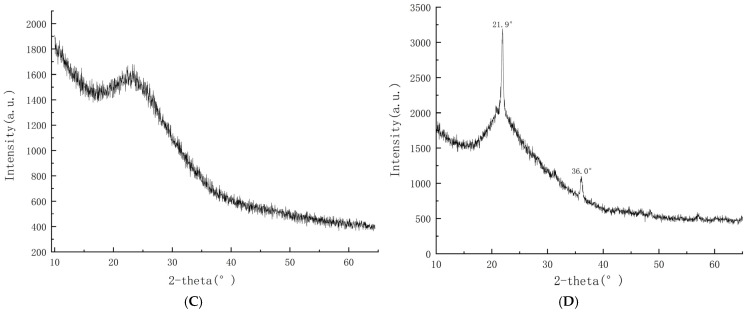
The XRD patterns of: (**A**) traditional TiO_2_ gel, (**B**) a mixture of nano-TiO_2_ and nano-SiO_2_ in a ratio of 1 to 8, mixed (**C**) hydrophobic SiO_2_–mixed TiO_2_ gel, and (**D**) nano-SiO_2_ powder.

In this study, the structures of traditional TiO_2_ gel and hydrophobic SiO_2_–mixed TiO_2_ gel were analyzed using X-ray diffraction (XRD). The obtained patterns of hydrophobic SiO_2_–mixed TiO_2_ gel, traditional TiO_2_ gel, mixture of nano-TiO_2_ and nano-SiO_2_ in a ratio of 1 to 8, and nano-SiO_2_ powder are displayed in Figure 6A–D. The XRD pattern of the traditional TiO_2_ gel in Figure 6A shows that several diffraction peaks were observed at 2θ of 25.3°, 37.7° 48.0°, 53.8°, 55.1° and 62.8°, which represents the (101), (004), (200), (105), (211), and (204) planes. This is because the TiO_2_ content in the ordinary colloid is relatively high, and the film prepared from this gel has undergone an annealing process that significantly affects the crystallization of TiO_2_ film materials [48]. As a result, more distinct peaks are shown in the X-ray diffractogram. Based on our results, the TiO_2_ in the film synthesized on low-iron crushed glass after annealing at 550 °C is a mixture of approximately 93–96% anatase and 4–7% rutile. Figure 6B,D shows the XRD images of both the mixture of TiO_2_ and SiO_2_ and only nano-SiO_2_ powder, which showed obvious characteristic peaks. The peak with a position angle of 21.9° and a value of 1221, as well as the peak with a peak position angle of 36.0° and a value of 310, belong to SiO_2_, and the other peaks in Figure 6B are characteristic peaks of TiO_2_ crystals.

For the hydrophobic SiO_2_–mixed TiO_2_ film, as presented in Figure 6C, the X-ray diffraction spectrum merely showed a broad diffraction peak at 2θ within the range of 20° to 30°, which represents the specific XRD peak of amorphous silica only. This is because the TiO_2_ content in the colloidal material used for preparing the hydrophobic SiO_2_–mixed TiO_2_ film is already relatively low, and hence neither the TiO_2_-related peaks nor any Ti-O-Si bonds could be observed. Additionally, since the high temperatures would destroy the hydrophobic property of the film, therefore, for the films formed solely by the hydrophobic SiO_2_–mixed TiO_2_ colloids, instead of the high temperature annealing process, we performed a 105 °C thermal treatment in an oven for 4 h. This was necessary for the adherence of the film, but ultimately led to the failure to generate a large amount of anatase TiO_2_ crystals. Consequently, there were no obvious characteristic peaks of titanium dioxide in the X-ray diffraction spectrum of the new gel, but only the specific XRD peaks of amorphous silicon were present. This also shows that there was neither a TiO_2_ crystal nor a SiO_2_ crystal in the hydrophobic gel, but only amorphous SiO_2_. Therefore, SiO_2_–mixed TiO_2_ is best used as a single-layer material coated on nine ordinary TiO_2_ films to provide hydrophobicity and a larger contact area, while ordinary TiO_2_ films can be fully crystallized by annealing every three layers, thus achieving the maximum anatase phase transformation and higher photocatalytic activity while being hydrophobic.

In addition, the BET (Specific Brunauer–Emmett–Teller) was measured using a specific surface area analyzer (Micromeritics Tristar 3000, Micromeritics Instrument Co., Norcross, GA, USA). The results were between 0.0561 m^2^/g for three layers of coating up to 0.0868 m^2^/g after nine layers of coating, which indicates that the higher the number of layers, the bigger the surface area will be.

#### 3.2.3. Contact Angle Test

**Figure 7:**
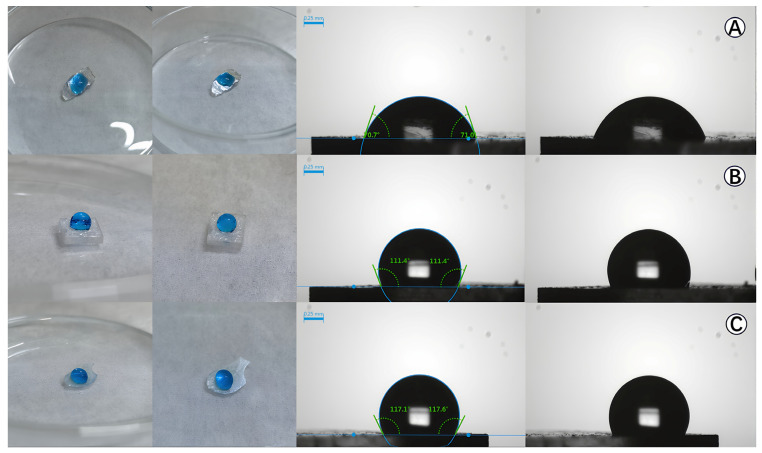
The hydrophobicity and hydrophilicity demonstration and water static contact angles of the three samples on both glass substrates (black) and crushed glass support surfaces (blue color droplets): (**A**) glass coated with three layers of ordinary TiO_2_ gel, (**B**) glass coated with three layers of hydrophobic SiO_2_–mixed TiO_2_ gel, (**C**) glass coated with two layers of ordinary TiO_2_ gel followed by one layer of hydrophobic SiO_2_–mixed TiO_2_ gel.

Contact angle testing is a common method for determining the hydrophobicity of material surfaces. θ_w_ is the water contact angle, known as the Young water contact angle. If θ_w_ < 90°, the surface is hydrophilic, and for θ_w_ > 90°, the surface is hydrophobic [49,50]. In this study, 5 mm × 5 mm glass substrates were used as the support, and three different coating processes were carried out on them to prepare three samples for contact angle testing. The measurements were repeated before and after the three rounds of the photocatalytic tests with no change in the outcomes. The results are shown in Figure 7.

From Figure 7, it can be observed that the water droplets containing methylene blue showed a spherical shape on the surface of the coated samples in Figure 7B (θ_w_ = 111°) and Figure 7C (θ_w_ = 117°), which were coated with the hydrophobic SiO_2_–mixed TiO_2_ gel. However, for Figure 7A, since it was coated with the conventional TiO_2_ gel, the droplets were flatter with a θ_w_ angle of around 71°, indicating that the material surface was hydrophilic.

### 3.3. Photocatalytic Removal of Methylene Blue

**Figure 8:**
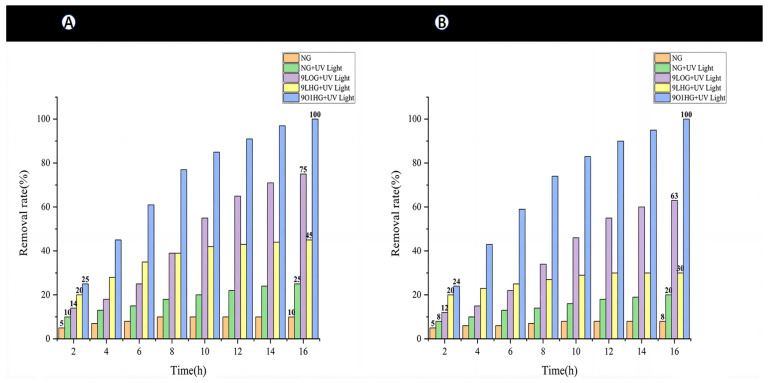
Comparison of methylene blue removal rates among different experimental groups: (**A**) the first round of the experiment and (**B**) the third round of the experiment.

Based on the quantitative analysis of methylene blue (MB) concentration using Equations (1) and (2), the degradation efficiency of various TiO_2_ film coatings was evaluated and summarized in Figure 8. The removal rate curves demonstrate that the 9O1HG coatings achieved the complete degradation of MB, reaching a 100% removal rate within 16 h. The 9LOG coating exhibited intermediate performance, attaining a removal rate of approximately 75% under the same conditions. The 9LHG coating was slightly insufficient, only achieving a removal rate of 45%.

To account for non-photocatalytic contributions in MB removal, the control experiments included an experimental group containing one NG and another with an NG under UV. The former showed a negligible removal rate of about 5%, attributable to the adsorption of MB on the glass surface. The latter exhibited a 25% reduction in MB concentration, indicating a combination of adsorption and limited photolysis of MB under UV. These control results confirm that the high degradation efficiencies observed for the TiO_2_-coated samples were predominantly due to photocatalytic activity rather than adsorption or photolysis.

The performance of the 9LHG coating can be attributed to the presence of numerous small voids on its surface, which absorbed more MB than the NG sample, as well as the partial photodegradation of MB. The superior performance of the 9O1HG coating can be attributed to its enhanced photocatalytic properties. The 9O1HG coating, comprising a combination of ordinary and hydrophobic TiO_2_ layers, may benefit from an optimized balance between pollutant adsorption capacity and photocatalytic ability. These research results ultimately indicate that only the common titanium dioxide film had a strong photocatalytic degradation ability for MB, while the hydrophobic film showed more enhanced absorption capacity for MB.

The 9LOG coating demonstrated a moderate initial removal rate of 75%, but its performance degraded continuously over the three cycles, dropping to 63% in the final round, accompanied by severe staining of the substrate. This suggests a lack of durability and a tendency for pollutant accumulation. The NG showed the lowest removal efficiency, which was primarily due to passive adsorption rather than any significant photocatalytic effect, as confirmed by its deep staining.

The photocatalytic performance of the 9LHG coating exhibited a notable decline over successive degradation cycles, with the methylene blue (MB) removal rate decreasing from 45% in the first round to 30% by the third. This reduction was attributed to the saturation of the coating’s adsorption capacity, which limited further uptake of MB molecules. Furthermore, 9LHG lacks intrinsic photocatalytic activity, preventing the degradation of adsorbed pollutants and resulting in the stabilization of removal efficiency at approximately 30% in subsequent cycles.

**Figure 9:**
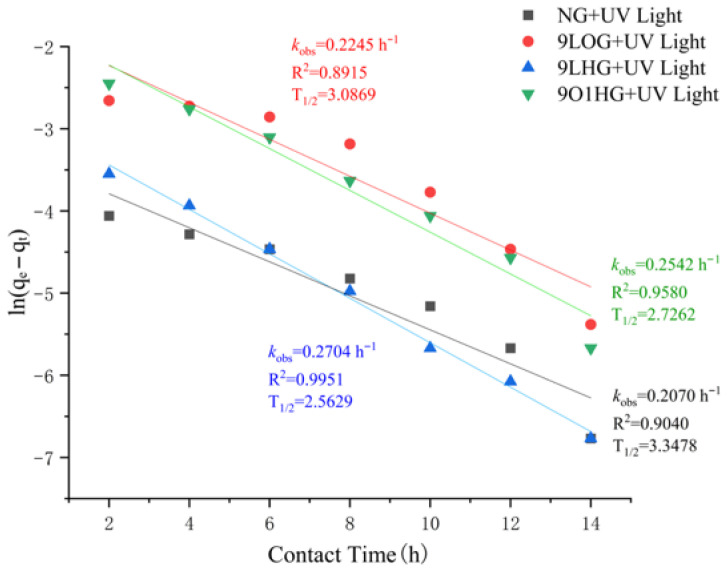
Kinetic plot of the PFO model for MB removal experiments using different samples.

To obtain a deeper and better understanding of the removal process and determine the optimal conditions for degrading methylene blue, a removal kinetic study using a linearized PFO model was performed to analyze the removal rate and half-life. Figure 9 presents the kinetic curve of the PFO model and the parameters for the removal of MB. It can be observed that the removal kinetics of MB can be well-simulated by the linearized PFO, and this was confirmed by the high R^2^ value. By comparing the reaction rate constants and half-lives, it was observed that the k values of 9LHG and 9O1HG were 0.2704 and 0.2542, respectively, which were significantly higher than those of NG (0.2070) and 9LOG (0.2245). Meanwhile, the half-lives of 9LHG and 9O1HG were 2.5629 and 2.7262, respectively, which were lower than those of NG (3.3478) and 9LOG (3.0869). The data clearly demonstrate that 9LHG and 9O1HG can remove MB more quickly. However, considering the removal rate of MB in the three-round experiments and the staining performance of the samples after the three-round tests, we can conclude that the reason why the removal rate of 9LHG is faster is largely due to the adsorption of MB on the surface of the samples, while 9LOG and 9O1HG can achieve the removal effect by the photocatalytic degradation of MB.

**Figure 10:**
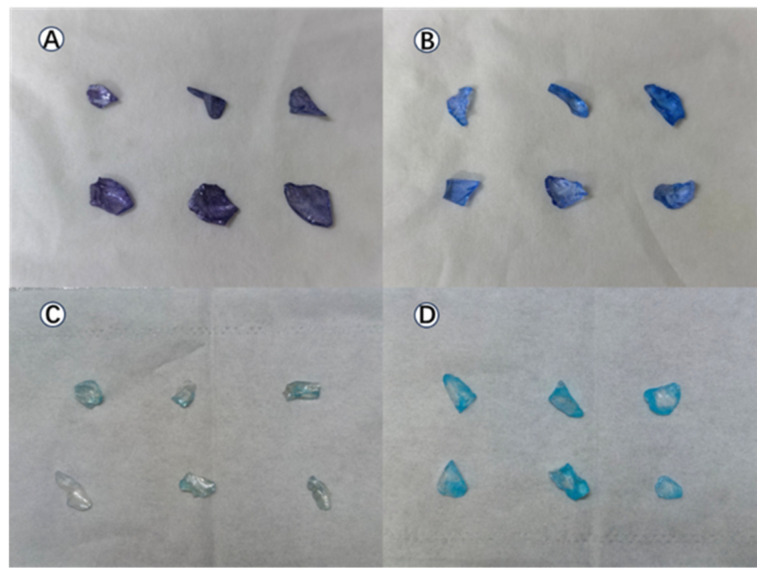
The degree of blue color staining on glass fragments: (**A**) 9LOG, (**B**) NG, (**C**) 9O1HG, and (**D**) 9LHG.

The varying degrees of staining across all samples after the three rounds of tests are visually shown in Figure 10. This figure presents a colorimetric assessment of the samples following three cycles of the methylene blue photocatalytic degradation experiment. A significant disparity in residual staining was evident among the four types of glass substrates. The 9LOG coating exhibited the most pronounced color change, developing a very dark, somewhat purplish hue. The NG coating also showed deep blue staining, indicating considerable adsorption of the dye on the sharp edges and rough surface of the crushed glass and poor self-cleaning efficacy. In contrast, the hydrophobic film 9O1HG demonstrated a marked reduction in blue intensity, confirming its enhanced resistance to fouling. While 9LHG also had hydrophobic properties, it lacked photocatalytic ability. As a result, continuous exposure to high concentrations of methylene blue caused the coloration to deepen. The anti-fouling performance of 9O1HG was superior to that of 9LHG. This can clearly be seen from the fact that its color was significantly lighter. This also indicates that both hydrophobicity and photocatalytic ability simultaneously affect the anti-fouling performance of the film.

## 4. Conclusions

In this study, a hydrophobic SiO_2_–mixed TiO_2_ gel was synthesized by a simple sol–gel method. Common titanium dioxide films and hydrophobic coatings were prepared on low-iron crushed glass by the dip coating method for degrading pollutants and preventing fouling and contaminant deposition on the coating surface to prolong the photocatalytic degradation ability.

SEM and XRD analyses revealed that the hydrophobic coating was amorphous, with no obvious cracks at low viscosity, and had uniformly distributed tiny pores, with enough anatase phase TiO_2_ particles. The BET results of nine layers of coating revealed an acceptable surface area. The contact angle test showed that the coating had an obvious stable hydrophobic performance. The methylene blue degradation experiment underpinned that when the hydrophobic coating was combined with the common titanium dioxide film, it demonstrated excellent photocatalytic degradation ability and acceptable anti-fouling performance. These analyses confirm that the film prepared by combining hydrophobic gel with ordinary gel not only has good photocatalytic performance, but also possesses hydrophobicity, avoiding the decline in photocatalytic performance due to pollutant deposition, which can play a better role in water pollutant degradation.

## Data Availability

The original contributions presented in this study are included in the article. Further inquiries can be directed to the corresponding authors.
